# Development of Real-Time Landmark-Based Emotion Recognition CNN for Masked Faces

**DOI:** 10.3390/s22228704

**Published:** 2022-11-11

**Authors:** Akhmedov Farkhod, Akmalbek Bobomirzaevich Abdusalomov, Mukhriddin Mukhiddinov, Young-Im Cho

**Affiliations:** Department Computer Engineering, Gachon University, Sujeong-gu, Seongnam-si 461-701, Korea

**Keywords:** face detection, emotion recognition, facial mask, landmark vectors application, facial expression detection

## Abstract

Owing to the availability of a wide range of emotion recognition applications in our lives, such as for mental status calculation, the demand for high-performance emotion recognition approaches remains uncertain. Nevertheless, the wearing of facial masks has been indispensable during the COVID-19 pandemic. In this study, we propose a graph-based emotion recognition method that adopts landmarks on the upper part of the face. Based on the proposed approach, several pre-processing steps were applied. After pre-processing, facial expression features need to be extracted from facial key points. The main steps of emotion recognition on masked faces include face detection by using Haar–Cascade, landmark implementation through a media-pipe face mesh model, and model training on seven emotional classes. The FER-2013 dataset was used for model training. An emotion detection model was developed for non-masked faces. Thereafter, landmarks were applied to the upper part of the face. After the detection of faces and landmark locations were extracted, we captured coordinates of emotional class landmarks and exported to a comma-separated values (csv) file. After that, model weights were transferred to the emotional classes. Finally, a landmark-based emotion recognition model for the upper facial parts was tested both on images and in real time using a web camera application. The results showed that the proposed model achieved an overall accuracy of 91.2% for seven emotional classes in the case of an image application. Image based emotion detection of the proposed model accuracy showed relatively higher results than the real-time emotion detection.

## 1. Introduction

The fast development of human–computer interaction and pattern recognition has brought significant convenience to humanity. Facial expression recognition (FER) is a crucial task for machines to understand the emotional well-being of people. FER is a powerful, natural, and universal signal that allows humans to convey their emotional states and intentions [1]. The range of FER applications is increasing, including online education, medical care, security, driving control, and other business sectors. The applications of FER in daily life will enable robots to understand the mental states and intentions of people based on their facial expressions and respond to them appropriately. In fact, human facial expression is one of the most pivotal ways for people to represent their emotional state. The main aim of expression recognition is to understand the inner thoughts of an individual regarding certain things or actions. For example, in certain countries, we can see the application of FER in facial expression recognition identification for capturing the fluctuating moods of elementary school students while in class to analyze their learning status and treat them as individuals based on their attitude. Alternatively, FER can be applied to judge the state of fatigue of pilots and drivers and to avoid traffic hazards through the technical implications. Therefore, in terms of inadvertently showing the true feelings of an individual, FER is more diverse than other communication methods [2]. From the technical side, FER extracts information representing the facial expression images with the help of computer image processing technology and then classifies the facial expression features according to human emotional expressions. Basic forms of expressions include sadness, happiness, fear, disgust, surprise, and anger.

Over the last few years, there has been rapid development in facial expression recognition technologies. FER research has mainly focused on feature extraction and classification. Facial expression features are extracted from facial regions, such as geometric and appearance features, which can be used for classification from input images or video streams [3,4]. Facial landmark analysis plays a crucial role in FER, including many applications derived from face processing operations and biometric recognition [5]. Based on the landmark implementation, we can analyze eye corners, eyebrows, mouth corners, etc., which enables us to come to certain facial expression conclusions with regard to the capture of the dynamic changes of facial features. The estimation of the feature vector to describe a person’s emotion is considered one of the foremost steps in facial expression identification. It is important to know the relative settings of the facial landmark points. To describe the movements of facial muscle landmarks, Yan et al. [6] defined facial landmarks as derivatives of action units (AUs). In 1971, Ekman [7] first divided expressions into six forms, and many studies have been based on emotion recognition studies relevant to defining facial features. Since AUs are suitable for FER, in [8,9], a facial expression analysis was conducted by computing the AUs using facial landmarks. A previous study [10] introduced a fusion approach based on landmarks and videos. The proposed models indicate that landmark features are effective for FER.

Herein, we present a graph-based representation of facial landmarks through a graph neural network GNN [11] for eye and eyebrow cases and propose an FER algorithm using a graph-based representation. In the first step of our proposed method, we built a model for facial expression recognition using the FER-2013 dataset. The model was trained using a non-masked face and facial expression identification. The second step of our research required the implementation of a facial expression recognition model weight to masked faces using transfer learning. Finally, we implemented the media-pipe face mesh algorithm to create landmarks on masked faces and then created emotional classes based on the facial expression recognition model.

The major contributions of this paper are as follows:

1. We propose a new GNN structure with landmark features as the input and output;
2. FER with more detailed input landmark modalities is applied by adopting an FER model by media-pipe face mesh algorithm;
3. Notably, this study proposes a two-fold contribution during expression recognition. That is, after the implementation of the face mesh algorithm on a masked face, the model detects facial expressions on either masked or non-masked faces.

**Figure 1:**
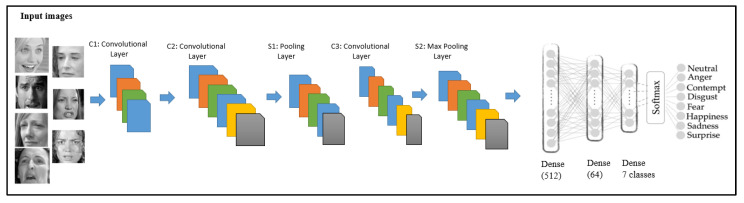
CNN architecture of the proposed method.

There have been many studies on FER with highly accurate results using convolutional neural network (CNN), as illustrated in Figure 1. Therefore, the main focus of this research is on masked face emotion recognition.

The paper is organized as follows: Section 2 reviews existing conventional studies on facial emotion recognition. Section 3 presents in detail an explanation of the landmark-based emotion recognition approach. The experimental results based on FER databases are discussed in Section 4. Section 5 describes specific shortcomings of the proposed method. Section 6 concludes the paper by giving an outline of our findings and upcoming research directions.

## 2. Related Works

### 2.1. Face Landmark Detection

Face detection is complicated owing to the different variability of human facial presence, such as pose, position and orientation, expression, complexion, frontal face objects (e.g., glasses, hairstyle, and beard), and external objects, such as differences in camera gain, lighting conditions, and resolution. Most researchers [12,13] have shown that precise landmarks are essential for achieving an accurate face recognition performance. Face detection is connected to image processing and computer vision interrelations with the instant detection of human faces. The first step in face recognition involves setting a face location in the image. In [14], face localization was conducted by finding the nose tip and then segmenting it by cropping the sphere centered at this tip. After face detection and segmentation, landmark localization is frequently used for face analysis. Many existing proposed techniques rely on the accurate localization of the corresponding landmarks or regions to achieve a rough alignment of meshes [15]. Landmark localization of facial features can be achieved by first locating the facial feature region of interest (RoI). Kakadiaris et al. [16] conducted face recognition with an annotated model that was non-rigidly registered for face meshes with an initial orientation of the face. There are several categories of facial landmark detection methods—holistic, co-strained local model (CLM), and regression-based [17] approaches. The most commonly used holistic method is the active appearance model (AAM) [18]. With regard to the CLM method, the most well-known model is the active shape model (ASM) [19]. Both models have several advantages. The ASM is more accurate in the case of point or contour localization and is less sensitive to fluctuations in illumination. Therefore, the ASM is relatively effective and suitable for applications that require precise contours. According to the anthropometric landmark distance measurements, the upper part of the facial key points contained only the eyebrows. The most likely landmark location approach treats the finding of a landmark as a two-class classification problem, such as a site, regardless of whether a location in an image is a landmark.

### 2.2. Classification of Facial Expressions

**Figure 2:**
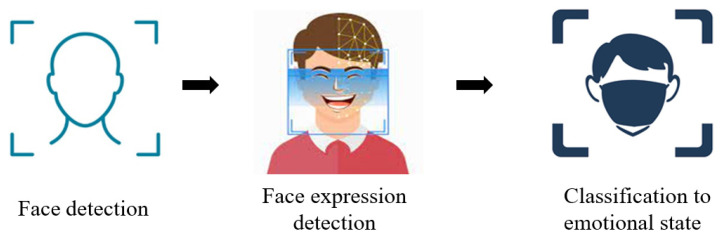
Facial expression recognition process.

Facial expressions can be easily observed and distinguished as a communication technique in the field of psychology [20]. Facial expressions provide information about a person’s emotions. FER analysis consists of three steps: (a) face detection; (b) facial expression detection; and (c) expression classification into an emotional state as shown in Figure 2.

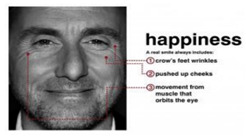
Happiness is a smiling expression that shows someone’s feeling of contentment or liking of something. A happy expression is classified as an upward movement of the cheek muscles and the sides or edges of the lips to form a smiling shape [7].
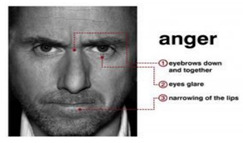
Anger is an expression of aggression. The characteristics of anger are a merging downward leaning of the inner eyebrows. The eyes become close to the eyebrows, the lips join, and the sides of the cheek lean downward [7]. 
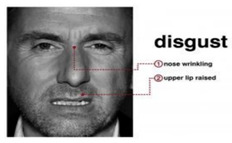
Disgust is an expression that shows a state of dissatisfaction with something or someone. An expression of disgust is classified when a person’s nose bridge between the eyebrows is wrinkled, and the lower lip goes down, showing the teeth [7]. 
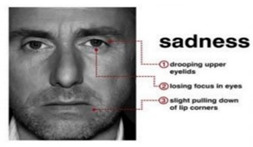
Sadness is an expression that represents disappointment or a feeling of missing something. Sadness is classified based on a lost focus of eyes, joined lips with the corners of the lips moving slightly downward, and a relatively wide distance between the eyes and eyebrows [7]. 
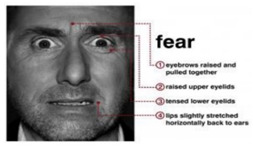
Fear is an expression that shows someone’s scarcity or fear of someone or something. The expression of fear is seen when eyebrows slightly go up, eyelids tighten, and lips are open horizontally along the side of the cheek [7]. 
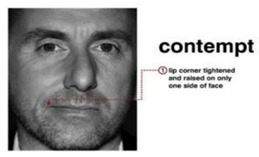
Contempt is an expression that shows no other expressions on the face, remaining neutral. Its characteristics are classified as a slight rise of one side of the lip corner [7]. 
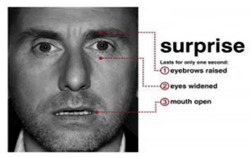
Surprise is an expression showing one’s excitement for a certain act. Its characteristics include a shocked face representation, raised eyebrows, open eyes, and an open mouth on the vertical side [7]. 

### 2.3. Face Emotion Detection

FER is a technology used to conduct a sentiment analysis of faces from different sources such as images and videos. Facial expressions are a form of nonverbal communication that provides hints of human emotions. In the early 1970s, psychologist Paul Ekman developed the Facial Action Coding System (FACS), which allows the interpretation of a person’s emotions by examining his/her facial expressions. These expressions are reported as a combination of isolated muscle movements, also referred to as action units (AUs) [21]. For example, the usual motion in a face expressing joy is claimed to be a smile, which is the result of tension in the symptomatic major muscle, classified as AU 12 or a “lip corner puller” based on the FACS [22]. Currently, big technological advancements, such as in the field of machine learning and pattern recognition, have played an outstanding role in the enlargement of FER technologies. Depending on the implementation of the algorithm, facial expressions can be grouped as basic emotions (e.g., anger, disgust, fear, joy, sadness, and surprise) or compound emotions (e.g., happy, happily surprised, sadly fearful, sadly angry, and sadly surprised) [23]. FER has gained special attention from researchers in the field of computer vision. Moreover, several companies offer their FER services through the web using an application programming interface (API), where users are able to send an image or video to their servers and obtain a specific data analysis of the defected facial expressions as a result [24]. One group of researchers proposed a facial recognition technique that uses histograms of oriented gradients (HOG) as descriptors and principal component analysis (PCA) along with linear discriminant analysis (LDA) as techniques for a dimensionality reduction of such descriptors [25].

### 2.4. Landmark-Based Emotion Recognition

In [26], a graph convolutional neural network is proposed to utilize landmark features for FER. Landmarks were applied to detect nodes, and the Delaunay method was used to build edges in the graph. In [27], a feature vector technique comprised three main steps in order to recognize emotions on masked faces. Researchers applied a landmark detection method to extract the features of occluded masked faces, and emotions were identified based on the upper facial landmark coordinates. In [28], a robust framework is presented for the detection and segmentation of faces, and landmark localization was applied to face meshes to fit the facial models. Landmark localization was conducted on the segmented faces to minimize the deviation of proposed technique from the mean shape. Similarly, researchers [29] used a mathematical technique to compare real-world coordinates of facial feature points with 2D points obtained from an image or live video using a projection matrix and Levenberg–Marquardt optimization. This technique was implemented to determine the Euler angles of the face and the best sets of facial landmarks. In addition, numerous studies using facial landmarks for face recognition, face emotion recognition, 2D- and 3D-based face detection, and other purposes have been conducted, as shown in Table 1..

## 3. Proposed Method

**Figure 3:**
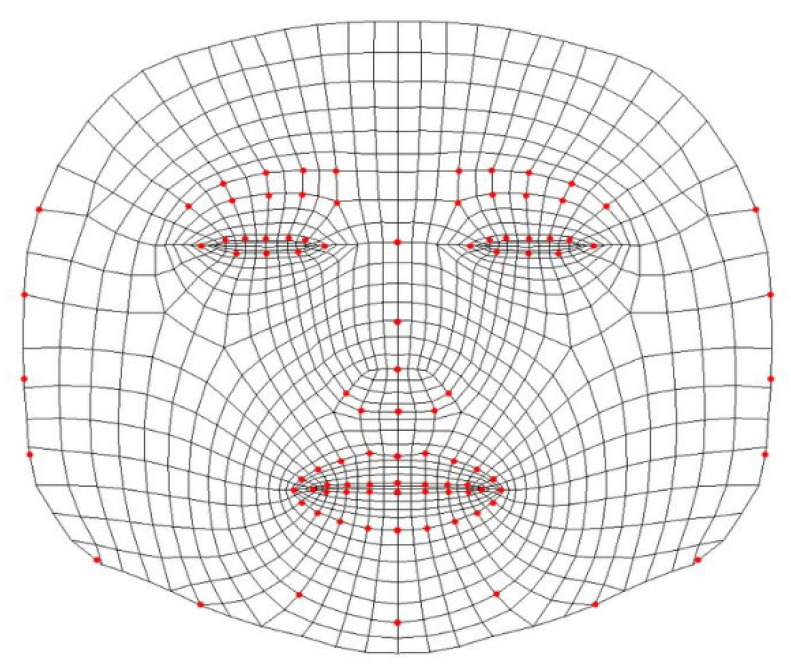
Media-pipe face-mesh mapping for 468 vertices.

The proposed method first applies facial identification and face emotion recognition steps on normal faces by using a Haar–Cascade classifier. The facial emotion recognition model was developed for faces. There has been a great number of studies on facial expression. However, as we mentioned, this paper focuses on the analysis of upper part facial expressions when people have a mask on their faces. For upper part facial landmarks, we gathered mainly eyes, eyebrows, landmarks disconnected to the nose and mouth. Figure 3 below represents seven emotional class landmark coordinates.

### 3.1. Haar–Cascade Classifier

Face detection is a popular subject area for researchers and offers a variety of applications. Face detection applications play a crucial role in surveillance systems as well as in security and biometric identification processes. The face detection process in this study used the Haar–Cascade classifier method. Motivated by the problem of face detection, the early Viola–Jones object detection framework, also popularly known as the Haar–Cascade algorithm, was first introduced in 2001 [33]. Haar denotes a mathematical function (Haar wavelet) in the form of a box. This algorithm identifies faces in an image or a real-time video. The calculation of the Haar value from a rectangular image is used to detect a vertical edge with darker pixels on its right and lighter pixels on its left. The Haar classifier was trained using Haar-like features by combining the integral graph method with the AdaBoost algorithm. The Haar-like features can reflect the gray-level change of an image because of the different features of the change in characteristics of the human face contrast. The Haar-like features process images in squares, where several pixels are represented. Every box then produces values that specify dark and light areas. Since the face representation in the image is automatically detected, the face position will adjust to the face data in each image. Moreover, there are Haar features that detect edges in other directions and structures by traversing the entire image. To search for particular features of an image, Haar-like features are continuously monitored from the top left of the image to the bottom right pixel by pixel. Researchers have also proposed an AdaBoost-based learning algorithm [34] to obtain an efficient classifier from the implementation of a small number of essential visual features. In other words, Haar-like descriptors are commonly used for texture descriptors. Haar–Cascade operates with grayscale images and does not work directly with image intensities [35].

### 3.2. Media-Pipe Model

**Figure 4:**
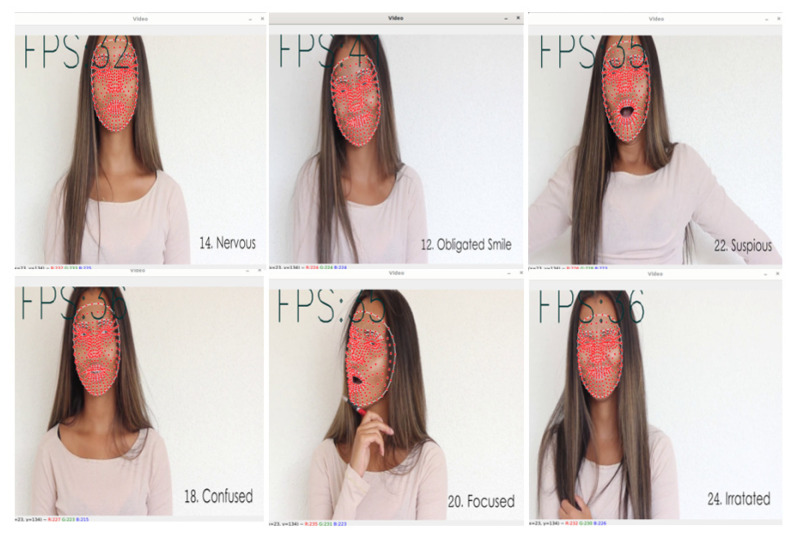
Media-pipe face-mesh application on video capture.

We developed a landmark detection method for faces with and without masks. In this stage, we adopted a landmark detection model for all masked and non-masked faces. In this step, the media-pipe framework was implemented to build machine learning pipelines. Media-pipe is a framework designed to build machine-learning pipelines for processing time-series data, such as video and audio. The media-pipe framework provides approximately 16 open-source pre-built examples based on specific pre-trained TensorFlow or TF-Lite models. The solution we implemented in our research is referred to as the media-pipe face-mesh model, which estimates 468 3D face landmarks in real time [36], as shown in Figure 3 and Figure 4.

Face-Mesh (FM) [37] is a face landmark-based machine learning model developed through transfer learning. FM was specifically designed to recognize a user’s facial topology in three dimensions. The machine learning architecture of FM was built on top of the Blaze Face (BF) [38] model. The main purpose of the BF model is to find faces in an image or video frame and make estimations in the bounding boxes. After the bounding boxes surround the face using BF, FM conducts an estimation in 3D coordinates. The pipeline consists of two real-time deep neural network models. The first is a detector that conducts operations on the full image and computations on the face locations. The second 3D face landmark model operates at these locations and predicts the approximate 3D surface through a regression.

### 3.3. Landmark Detection

Obtaining the region of interest (RoI) of both the right and left eyes enables the extraction of the feature points corresponding to the eyes. Each landmark localization in the facial muscles presents a strong relationship with other specific landmarks that are placed in a similar position or connected muscles. It was found that the landmarks of the external region negatively affected facial emotion recognition performance. Therefore, to increase the performance of the model, we used the media-pipe face mesh model to detect landmarks for the eyes and eyebrows, where landmarks were input features:

LM = {x_t,p_, y_t,p_ | 1 ≤ t ≤ T, 1 ≤ p ≤ P}. (1)

Here, LM indicates a set of landmarks, and (x_t,p_, y_t,p_) are the 2D coordinates of each landmark, where P and T represent the number of landmarks and frames, comparatively.

### 3.4. Gaussian Processes

From probability theory and statistics, a Gaussian process (GP) is considered a collection of random variables indexed by time or space. In the proposed model, an infinite collection of scalar random variables is the input space between landmark key points for any finite set of inputs X = {x_1_, x_2_, …, x_n_}, where the random variables fΣ[f(x_1_), (x_2_), …, f(x_n_)] are allocated with regard to a multivariate Gaussian distribution f(X)–GP(m(x), k(x,x′)) [39]. The GP is specified by the mean function m(x) = E[f(X)] and a covariance function given by:

k(x,x′) = E[(f(x) − m(x) × (f(x′) − m(x′))^T^]. (2)

We defined the landmark key points as a vertex covariance matrix through location information. For edge construction, the Delaunay method was implemented [40]. Each vertex represents a 2D feature vector, as shown in the following equations:

V = {v_t,p_ | 1 ≤ t ≤ T, 1 ≤ p ≤ P} (3)

v_t,p_ = [x_t,p_, y_t,p_]. (4)

The Delaunay technique constructs triangular meshes among all landmarks [38] and is an efficient method for analyzing facial emotions [41,42]. While the mesh composition only indicates whether edges are connected, we include the squared exponential kernel, i.e., radial basis function (RBF) kernel, as shown below:

k(x,x′) = σ^2^_f_exp(−0.5l^2^(x − x′)^2^), (5)

where σ^2^_f_ represents the variance of the functions, and l^2 i^ndicates the length of the scale of any two uncorrelated inputs (x_i_, x_j_).

Thereafter, multiplication is included to show the length of scale (l^2^) of the distance to represent the strength of the edges, as follows:

C = {e_t,i,j_|1 ≤ t ≤ T, 1 ≤ I, j ≤ P} = {|v_t,i_ − v_t,j_|1 ≤ t ≤ T, 1 ≤ i, j ≤ P} (6)

F = DM({x_t,p_ × y_t,p_|1 ≤ t ≤ T, 1 ≤ i, j ≤ P}) = {|a_t,i,jj_|1 ≤ t ≤ T, 1 ≤ i, j ≤ P}, (7)

where DM represents the Delaunay method, and F depicts the adjacency matrix that contains binary values. Subsequently, V and C are the compositions of 2D vectors and scalar values that comprise a graph structure.

G = (V,C),(8)

where G indicates the geometric information of the facial emotions. Since we defined how to classify facial emotions, we trained the proposed model by first using the Haar–cascade classifier to detect faces, implemented media-pipe face mash landmark detection on the faces, and finally developed seven emotional classes for that model.

## 4. Experiments and Results

In this section, we present the implementation of the proposed method using machine learning and deep learning tools.

### 4.1. Dataset

**Figure 5:**
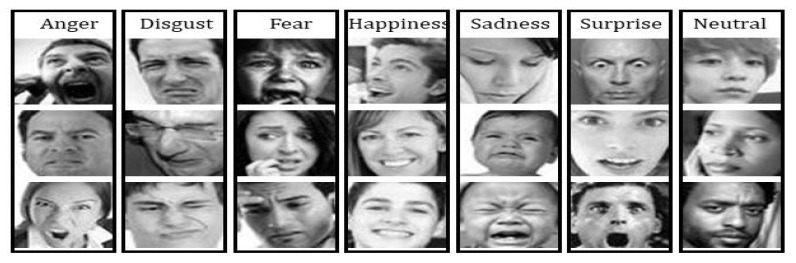
FER2013 dataset representation for seven emotional classes.

Based on the purpose of this study, the first step was collecting the dataset. We applied the 2013 Facial Expression Recognition dataset (FER-2013), which is available on Kaggle.com. The FER-2013 dataset was introduced at the International Conference on Machine Learning (ICML) in 2013 [43] by Pierrol and Aaron. The dataset consists of 35,887 images, with seven different types of facial expressions, as shown in Figure 5.

To detect emotion, we needed a face classifier to determine whether face features exist. The Keras, Tensorflow, and OpenCV tools were applied to train the model using the FER 2013 dataset. In the model development, 24,176 images were used for the training set, and 3006 images were used for the validation set. There were seven classes, i.e., Happy, Angry, Disgust, Fear, Sadness, Surprise, and Neutral. Each figure was composed of a grayscale image with a fixed pixel resolution of 48 × 48 (Table 2).

**Figure 6:**
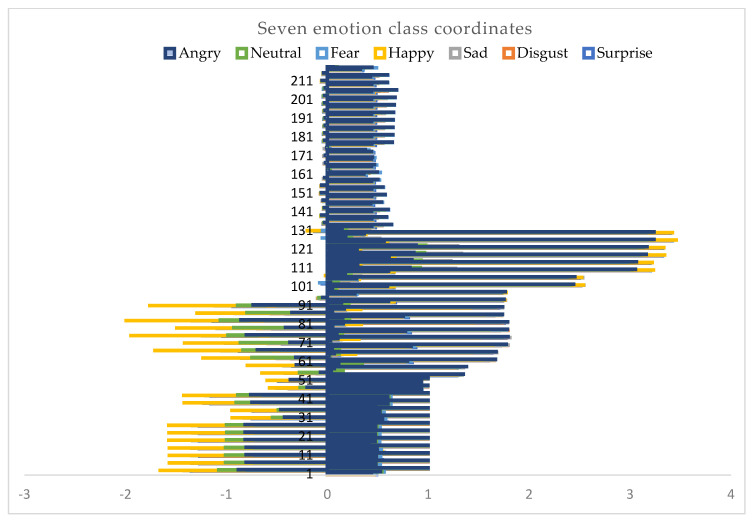
Seven emotion class landmark coordinates of facial key points.

To train the model with only eye- and eyebrow-based landmarks, we first gained weights for the emotion detection model. We trained the Haar–cascade classifier to detect faces and emotions. After detection, we captured coordinates of emotional class landmarks and exported to a comma-separated values (csv) file in seven emotional classes. After the emotion detection model was trained, we applied landmarks of the eyes and eyebrows and specified emotional classes to that model. Landmarks were adjusted to relative emotional classes. In Figure 6 we can see some relevant landmark points for seven emotional classes. The model was trained on a multi-class classification model in order to understand the relationship between emotional classes and representative coordinates.

**Figure 7:**
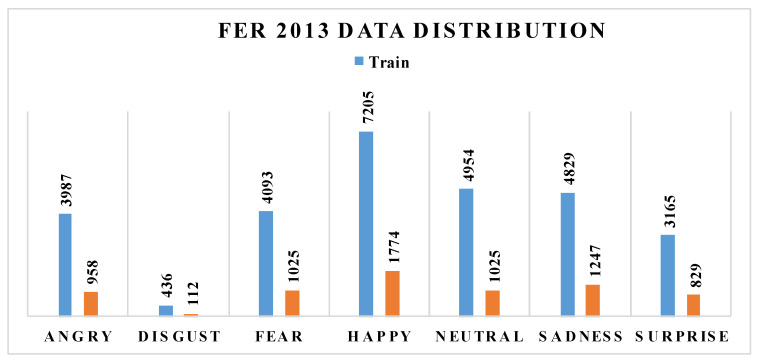
Dataset distribution for emotion recognition research.

Figure 6 depicts more than two hundred facial landmark coordination of facial key-points on seven emotional classes. Figure 6 presents a small set of examples to show how emotional class coordinates represent in between minus three (−3) and four (4) in x axes. In real testing, the case model will make a prediction based on thousands of emotion class coordinates, as shown in Figure 7.

### 4.2. Pre-Processing the Model

Table 3 below is a representation of a convolutional neural network development that is specialized to detect emotional classes of the human face. The convolution layer is the core of the CNN used to represent the characteristics of a local connection and value sharing. The input image and several trainable convolution filter algorithms were implemented to produce the C1 layer, including the batch normalization technique, a rectified activation function (ReLU) activation function, and max pooling parameters, which were also implemented in the first layer of the emotion recognition model.

The batch normalization technique was used to standardize the inputs to a layer, stabilize the learning process of the algorithms, and save more time by reducing the number of training epochs. Subsequently, ReLu was applied. Without the activation function, our model behaves as a linear regression model. Since our model was trained in the case of an image dataset, ReLU allowed the network to learn complex patterns in the data. Mathematically, the ReLU is expressed as follows:

f(x) = max(0,x).(9)

Next, a max-pooling operation was applied to calculate the maximum value in each patch of the facial feature map. The pooling operation involves sliding a two-dimensional filter over each channel of the feature map and reducing the number of dimensions of the feature map. The pooling layer summarized the features present in a region of the feature map generated by the convolution layer, and operations were then conducted based on the summarized features instead of precisely positioned features. This process of dimensionality reduction makes the model more robust to variations in the positions of the features in the input image.

After the convolution and pooling operations were applied to the input, the model was sufficiently small and adjusted to high-level features. The last layer of the proposed CNN used a softmax classifier, which is a multi-output competitive classifier, as given in Table 3 It provided the probability of the input belonging to one of the possible outcomes with regard to the labeled classes of the dataset. When every sample was an input, every neuron made an output in a value range between 0 and 1. Depending on the value ranges of input data, the model made a probability prediction of the labeled classes.

### 4.3. Evaluation Metrics

The general acceptance of agreement with true facts can be evaluated based on the computation of correctly recognized class numbers (true positives—TP), the number of correctly recognized examples that do not belong to the class (true negative—TN), and examples that either were incorrectly assigned to the class (false positive—FP) or that were not recognized as class examples (false negative—FN), as in our previous studies [44,45,46,47,48,49]. The number of samples in each combination of example classes and prediction classes was then summarized in the sample of confusion matrix in the metrics of TP, TN FP and FN.

(10) $$\mathrm{Sensitivity},\;\mathrm{the}\;\mathrm{true}\;\mathrm{positive}\;\mathrm{rate}\;(\mathrm{TPR}),\;\mathrm{or}\;\mathrm{recall}=\frac{\mathrm{TP}}{\mathrm{TP}+\mathrm{FN}}$$

(11) $$\mathrm{Specificity}\;\mathrm{or}\;\mathrm{False}\;\mathrm{Positive}\;\mathrm{Rate}\;(\mathrm{FPR})=\frac{\mathrm{TP}}{\mathrm{FP}+\mathrm{TN}}$$

(12) $$\mathrm{Precision}=\frac{\mathrm{TP}}{\mathrm{TP}+\mathrm{FP}}$$

(13) $$F-1\;\mathrm{score}=\frac{2*\mathrm{Recall}*\mathrm{Precision}}{\mathrm{Recall}+\mathrm{Precision}}.$$

### 4.4. Proposed Model Performance

Table 4 shows the performance of the proposed emotion detection model for the seven emotion classes. Our research suggests that it is more difficult to recognize an individual’s emotional state when a mask covers their mouth and nose than when it does not. Consistent with our prediction, we found that the accuracy with which people could identify an expression on a masked face was lower for all the emotions we studied (anger, disgust, fear, happy, neutral, sad, and surprise expressions). The performance of the proposed emotion recognition for seven emotion classes is shown in Table 4. The emotions of happiness and surprise achieved the highest precision based on the fact that people’s eyebrows and eyes shift and change more in situations of joy and wonder—0.85 and 0.78, respectively. In contrast, in the cases of fear, anger, and sadness, the landmark contours of the eyebrows and eyes did not change much; therefore, 0.50, 0.53, and 0.54 precision was achieved, respectively. Furthermore, 0.69 precision was achieved even though eyebrows and eyes in disgust are similar landmark contours to the fear emotion.

**Figure 8:**
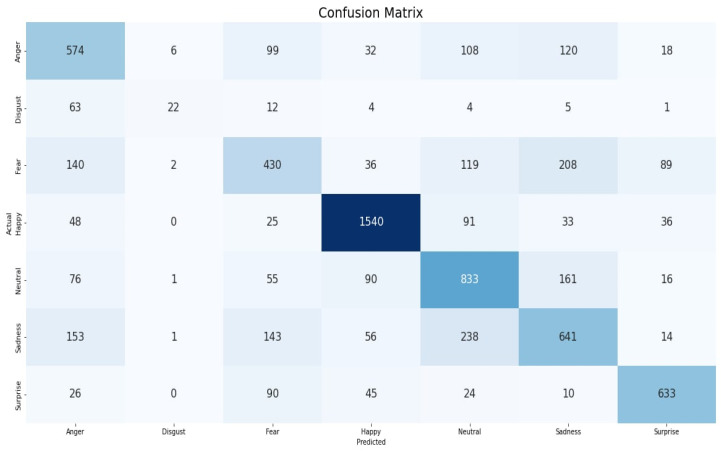
Confusion matrix of proposed method for the FER-2013 dataset.

Figure 8 below allows visualization of the performance metrics of the proposed method in seven emotional classes in a comparison of “actual” and “predicted” sets. Based on the numbers represented in Figure 8, we can analyze the values of TP, FP, TN and FN.

**Figure 9:**
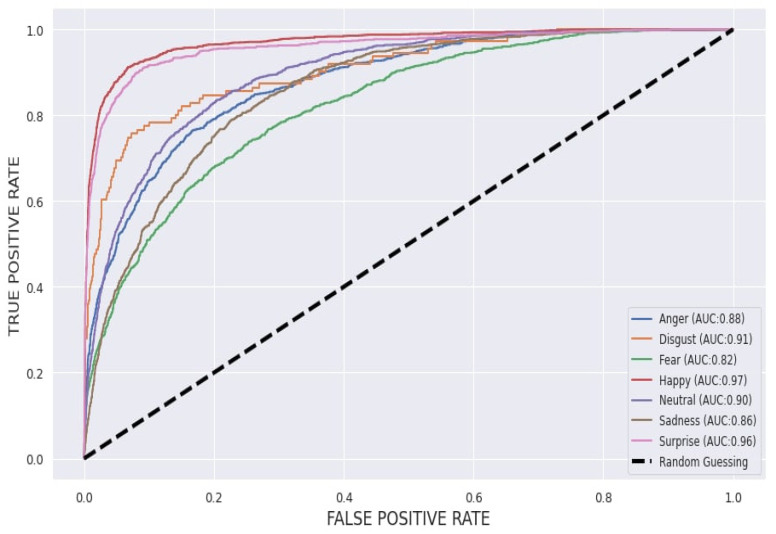
Classification performance depicted as ROC curves and corresponding area under the curve (AUC) for the overall emotion recognition performance when applying the FER-2013 dataset.

A receiver operating characteristic (ROC) curve was created by plotting the TPR against the FPR, as illustrated in Figure 9.

The ROC curve shows the performance of the classification model at all classification thresholds by plotting the TPR and FPR parameters. Plots of the four results (TP, FN, FP, and TN) in the ROC space are shown in the figure. Evidently, the result of the real Happy class shows the highest predictive accuracy among other emotional classes. As compared to the ROC curve analysis, based on Table 5 the Happy, Surprise, and Disgust emotion classes are classified as perfect with a range of 97%, 96%, and 91%, respectively. This indicates that the facial expression recognition of our proposed model is more uniform than that of the other emotional classes. The other emotions, such as the Neutral, Anger, and Sadness classes, were comparatively low in their range, reaching 90%, 88%, and 86%, respectively.

**Figure 10:**
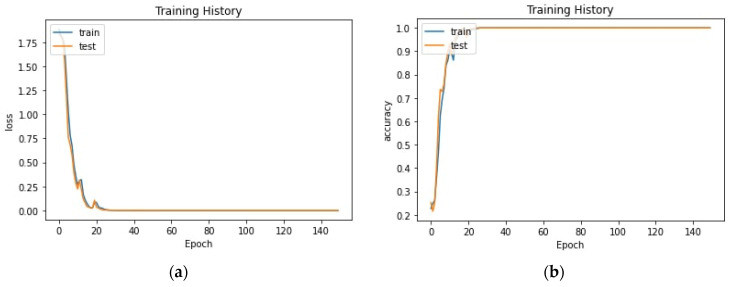
Loss (**a**) and accuracy (**b**) of proposed model in training and testing history of 150 epochs.

In Table 5, the proposed model is compared with other models, showing an outperforming recognition rate. Table 5 shows the evaluation of our prediction model against the actual data. We checked the model evaluation in three varied classification models, such as linear regression, random forest and gradient boosting. Figure 10 shows loss and accuracy of proposed model in training and testing history of 150 epochs.

**Figure 11:**
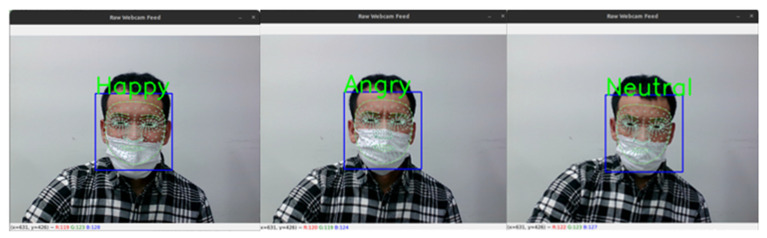
Haar-cascade face detection based real-time emotion recognition video capture.

To evaluate the qualitative performance of the proposed method, a practical live video analysis was performed. The model performed two detections, such as class and the probability percentage of the model. Figure 11 depicts Haar-cascade based face detection and emotion detection without probability estimations.

**Figure 12:**
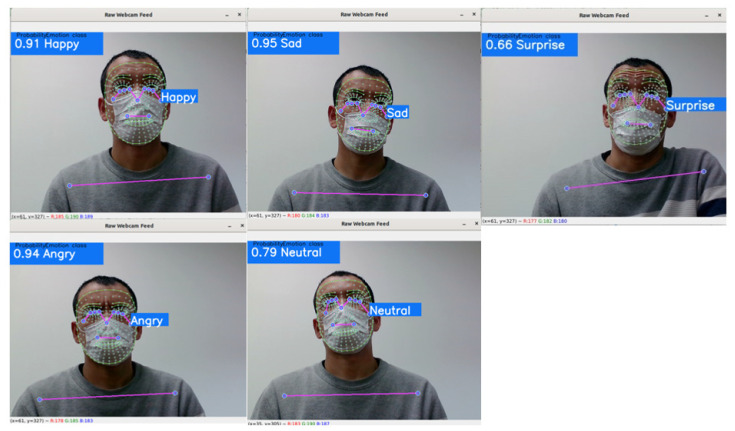
Real-time training results of proposed model in five emotional classes.

The recognition percentage of the emotional class and emotional class name is shown in the top left corner of the web camera and on the right side of the face. Figure 12 depicts the model’s performance in the real-time emotion analysis. Captures were taken for five emotional classes when the model reached its best detection percentage. Results indicate that, in real-time, the emotion analysis model achieved relatively higher percentages when landmark contours vary significantly.

## 5. Limitations

**Figure 13:**
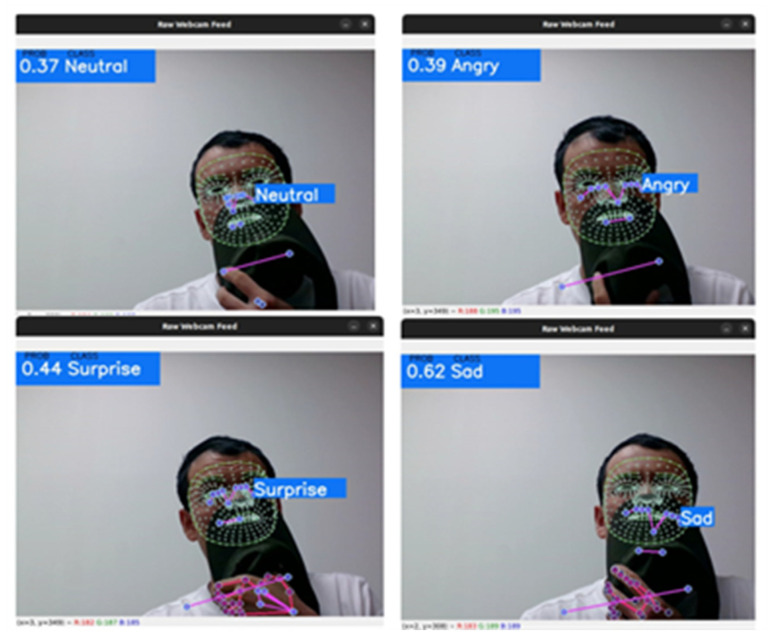
Low accuracy results of proposed model in real-time emotion recognition.

Since the model applies FER based only on the upper facial landmarks, lower facial landmarks are still represented as a building bias in our model, as shown in Figure 13. In a future study, we will improve the model by removing the lower facial landmark representations. Further improvements will be made with the collaboration of researchers [53,54,55,56] in detecting face color and iris change impact on emotion detection.

## 6. Conclusions

Overall, the Haar–Cascade classifier implemented on a CNN enables the detection of faces and emotion recognition, and we used this classifier in the development of an FER model using non-masked faces. Next, transfer learning was implemented to transfer the pre-trained FER model, and we applied the media-pipe face mesh model to adjust the landmarks based on the trained model. Finally, when we ran the developed CNN model, it automatically classified facial emotions even when masks covered the faces. After comparing with the models in recent years, our proposed approach has achieved good emotion detection results in image-based experiments. A model comparison shows a 90% overall accuracy compared to the R-CNN, FRR-CNN, and CNN-edge detection algorithms. Although our model has achieved relatively high results in image-based emotion identification, real-time emotion detection showed lower accuracy results because of the biases and noises in the facial expressions. In our further research, we will focus on achieving high emotion detection on masked faces in real life by overcoming biases and noises, such as the image being too dark, blurred or other external factors.

Future tasks include solving blurry problems under dark conditions and increasing the accuracy of the approach. We plan to develop a small real-time model with a reliable landmark-based emotion recognition performance using 3D CNN, 3D U-Net and YOLOv environments [57,58,59,60,61,62,63].

## Figures and Tables

**Table 1:** Comparison of feature parts and landmark application, focus areas.

Methods	Input Feature Parts (Upper: Eyes, Eyebrows Lower: Nose, Mouth)	Landmark Application Area	Focus Area
RTFER [30]	Upper + Lower	Face landmarks	Mainly focus to Lower
ResiDen [31]	Upper + Lower	Face landmarks	Mainly focus to Lower
DenseNet [32]	Upper + Lower	Face landmarks	Mainly focus to Lower
Proposed method	Upper	Face landmarks	Mainly focus to Upper

**Table 2:** FER-2013 dataset distribution for training and testing sets on seven emotional classes.

	Angry	Happy	Sad	Neutral	Fear	Disgust	Surprise
Train	3987	7205	4829	4954	4093	436	3165
Test	958	1774	1247	1233	1025	112	829

**Table 3:** CNN layers for emotion recognition.

Layer (Type)	Output Shape	Parameter Numbers	Activation
conv2d (Conv2D)	(None, 48, 48, 32)	320	ReLU
conv2d_1 (Conv2D)	(None, 48, 48, 32)	9248	ReLU
max_pooling2d (MaxPooling2D)	(None, 24, 24, 32)	0	ReLU
conv2d_2 (Conv2D)	(None, 24, 24, 64)	18,496	ReLU
conv2d_3 (Conv2D)	(None, 24, 24, 64)	36,928	ReLU
max_pooling2d_1 (MaxPooling 2D)	(None, 12, 12, 64)	0	ReLU
conv2d_4 (Conv2D)	(None, 12, 12, 128)	73,856	ReLU
conv2d_5 (Conv2D)	(None, 12, 12, 128)	147,584	ReLU
max_pooling2d_1 (MaxPooling 2D)	(None, 6, 6, 128)	0	ReLU
flatten (Flatten)	(None, 4608)	0	None
dense (Dense)	(None, 512)	2,359,808	ReLU
dense_1 (Dense)	(None, 64)	32,832	ReLU
dense_2 (Dense)	(None, 7)	455	Softmax

**Table 4:** Classification report of emotional classes.

	Precision	Recall	F1-Score	Support
Anger	0.53	0.60	0.56	957
Disgust	0.69	0.20	0.31	111
Fear	0.50	0.42	0.46	1024
Happy	0.85	0.87	0.86	1773
Neutral	0.59	0.68	0.63	1232
Sad	0.54	0.51	0.53	1246
Surprise	0.78	0.76	0.77	828
Accuracy			0.65	7171
Macro avg	0.64	0.58	0.59	7171
Weighted avg	0.65	0.65	0.65	7171

**Table 5:** Comparison of the proposed method with other algorithms, and classifier results.

Method	Recognition Rate (%)	Proposed Method with Four Different Classifiers (%)	Classification Rate (%)
R-CNN algorithm [50]	79.34	Linear regression	99.04%
FRR-CNN algorithm [51]	70.63	Random forest	99.8%
CNN-Edge detection method [52]	88.86	Gradient boosting	99.42%
Our proposed method	91.2		

## Data Availability

Not applicable.
